# The Impact of Positive Childhood Experiences: A Systematic Review Focused on Children and Adolescents

**DOI:** 10.1177/15248380251320978

**Published:** 2025-02-28

**Authors:** Marta Sousa, Ana Beatriz Machado, Marina Pinheiro, Bárbara Pereira, Sónia Caridade, Telma Catarina Almeida, Ana Rita Cruz, Olga Cunha

**Affiliations:** 1 University of Minho, Braga, Portugal; 2 ISPA – Instituto Universitário, Lisbon, Portugal; 3 Egas Moniz School of Health & Science, Caparica, Almada, Portugal; 4 Egas Moniz Center for Interdisciplinary Research (CiiEM), IUEM, Caparica, Portugal; 5 LabPSI – Laboratório de Psicologia Egas Moniz, IUEM, Caparica, Portugal; 6 Lusófona University, Lisbon, Portugal

**Keywords:** positive childhood experiences, adverse childhood experiences, benevolent childhood experiences, counter-aces, children, adolescents

## Abstract

Childhood and adolescence are crucial stages of life, characterized by significant changes that profoundly influence overall development. While positive childhood experiences (PCEs) can help mitigate the effects of adverse events during these formative years, they have not been as thoroughly researched. Then, this systematic review aims to address this gap by organizing the existing literature on PCEs and examining their impact on both positive and negative outcomes in children and adolescents. A search through databases such as B-On, PsycINFO, PubMed, SCOPUS, and Scielo, as well as supplementary searches, identified 30 studies that met the inclusion criteria. The results indicate that most studies were published in the last 4 years, primarily in the USA, and focused on community populations with mixed samples. In addition, the results reveal that among children and adolescents, higher levels of PCEs were associated with better mental health outcomes (e.g., reduced depressive symptoms, anxiety, self-harm, substance use, and suicidal ideation), improved psychosocial outcomes (e.g., enhanced adult functioning and future orientation), better academic achievement (e.g., reduced absenteeism and fewer academic difficulties), and some improvements in physical health (e.g., reduced chronic pain). However, the relationship between PCEs and behavioral outcomes showed mixed results. Strengthening efforts to promote PCEs and resources that support child and adolescent resilience is crucial. Further research involving diverse samples is needed to gain a deeper understanding of the role of PCEs.

Childhood and adolescence are life stages marked by significant changes that profoundly influence individuals’ overall growth and maturation. During these formative years, cognitive, emotional, and social competencies are being shaped (Branje et al., 2022; Pozzi et al., 2021). Research indicates that these years are also associated with an increased susceptibility to mental health issues (Scully et al., 2020) and behavioral problems (e.g., Bevilacqua et al., 2021). Specifically, symptoms such as anxiety and depression (Bellis et al., 2013), as well as temper tantrums, attention deficit hyperactivity disorders, and oppositional, defiant, or conduct disorders (Ogundele, 2018) are more likely to emerge during this period. Studies showed that early onset mental and behavioral disorders are often linked to lifelong challenges (Mulraney et al., 2021; Ogundele, 2018).

These outcomes are often associated with both adverse childhood experiences (ACEs) and positive childhood experiences (PCEs; Gaspar et al., 2012; Lukoševičiūtė et al., 2022). However, while ACEs have been extensively studied, PCEs have received comparatively less attention. Only recently has the scientific community begun to explore the role of PCEs, particularly their interaction with ACEs, with most research focusing on adulthood outcomes (Cunha et al., 2025; Han et al., 2023). Therefore, understanding how PCEs influence various outcomes in childhood and adolescence, regardless of the presence of ACEs, is crucial for expanding our understanding of PCEs and developing effective prevention and intervention strategies.

## Adverse Childhood Experiences

ACEs are potentially traumatic events that occur during childhood and can profoundly affect development, health, and overall well-being (Boullier & Blair, 2018; Dube et al., 2006). ACEs encompass various forms of abuse (physical, emotional, and sexual), neglect (physical and emotional), and household dysfunction, such as witnessing domestic violence, parental substance use or mental health disorders, and parental separation or divorce (Boullier & Blair, 2018; Dube et al., 2006; Felitti et al., 1998). Moreover, the prevalence of ACEs is higher than in the community, but there are disparities in their prevalence (e.g., Madigan et al., 2023; Vitopoulos et al., 2019). Research consistently shows that individuals from minoritized racial and ethnic groups experience a higher prevalence of four or more ACEs compared to others (Madigan et al., 2023). This disparity is particularly notable in populations identifying as Indigenous or Native American, as they demonstrate significantly higher rates of exposure compared to those identifying as White or Asian (Madigan et al., 2023). Furthermore, these percentages are even higher in forensic populations, underscoring the compounded vulnerabilities in these contexts (Vitopoulos et al., 2019).

Apart from their prevalence, these experiences are strongly associated with numerous long-term negative outcomes, including potential harm to brain development (Anda et al., 2006; Boullier & Blair, 2018). Research shows that ACEs often accumulate throughout childhood (Kessler et al., 2010) and are strongly linked to socioeconomic disadvantages, lower educational attainment, and employment challenges in adulthood (Dube et al., 2010). Moreover, the presence of multiple ACEs is associated with various negative outcomes, even in the early years of life, namely the presence of externalizing problems (McDoniel & Bierman, 2023) and poor physical health outcomes (Liming & Grube, 2018).

## Positive Childhood Experiences

In the literature, the terms PCEs Benevolent Childhood Experiences (BCEs), and counter-ACEs are often used interchangeably, referring to positive experiences and protective factors that contribute to well-being and healthy development during childhood (Cain et al., 2024). These experiences encompass stable home environments, nurturing family relationships, predictable routines, supportive relationships at school, and enriching experiences in the community (Almeida et al., 2021; Crandall et al., 2019; Narayan et al., 2018). Research highlights the role of PCEs as promotive and protective factors (Crandall et al., 2019; Narayan et al., 2018), buffering against the negative effects of childhood adversity (Almeida et al., 2023). PCEs also mitigate the harmful effects of ACEs, especially in populations facing extreme adversity (Almeida et al., 2023). Strong evidence links PCEs to enhanced resilience, emotion regulation, empathy, and overall well-being (Crandall et al., 2019; Narayan et al., 2018).

PCEs are increasingly acknowledged as crucial for fostering healthy emotional, cognitive, and behavioral development, particularly in mitigating the negative impact of ACEs (Almeida et al., 2023; Crandall et al., 2019; Masten & Cicchetti, 2016). The concept of “allostasis” is key to understanding the long-term impact of PCEs (Boullier & Blair, 2018). Allostasis refers to physiological adaptation to stress, allowing the organism to maintain stability despite environmental changes (Boullier & Blair, 2018). Stress usually initiates a recovery period that restores normal functioning, but protective factors, such as PCEs, can strengthen this regulatory process and foster resilience in children (Boullier & Blair, 2018).

Furthermore, like ACEs, PCEs are cumulative, with their benefits amplifying across multiple domains and fostering positive adjustment and adaptation (Han et al., 2023). This cumulative effect underscores their role in supporting favorable developmental trajectories (Crandall et al., 2019; Evans et al., 2013; Masten & Cicchetti, 2016; Masten et al., 2021). PCEs, even in the presence of ACEs, have been shown to buffer the long-term effects of adversity and support normative functioning across domains (Gartland et al., 2019; Toth & Cicchetti, 2013).

## Resilience Models

Three resilience models have been proposed to explain how protective factors interact with risk factors. In the compensatory model, positive factors are believed to have an independent beneficial effect on outcomes, regardless of risk exposure, indicating that these factors exert their influence without being affected by the presence of risk (Andersson & Ledogar, 2008; Bunting et al., 2023). On the other hand, the protective model suggests that protective resources can moderate or diminish the impact of risk factors on negative outcomes. These protective factors may either neutralize or weaken the adverse effects of risks or amplify the positive effects of other promotive factors, thereby improving the outcome (Feming & Ledogar, 2008). The challenge model presents a different perspective, where the relationship between a risk factor and an outcome is curvilinear. According to this model, both low and high levels of exposure to a risk factor lead to negative outcomes, while moderate levels of risk are associated with less negative or even positive outcomes. This moderate exposure to adversity can serve as a form of “inoculation,” helping individuals build resilience against future adverse exposures and teaching them how to overcome challenges (Bunting et al., 2023; Feming & Ledogar, 2008).

Recent empirical evidence supports these models, particularly the compensatory model, through research linking PCEs to reduced mental health problems despite adversity (Kocatürk & Çiçek, 2023). For example, Crandall et al. (2020) demonstrated that while ACEs increased the likelihood of mood and anxiety disorders, self-harming behaviors, and suicidal ideation in adolescents, the presence of PCEs correspondingly reduced these risks. This suggests that PCEs independently promote mental health, aligning with the compensatory model’s framework.

Further research has demonstrated that pre-adolescent children who faced cumulative discrimination were at heightened risk for internalizing and externalizing behaviors (Bethell et al., 2019; Glickman et al., 2021). However, PCEs were found to mitigate this risk, reducing the association between cumulative adversity and these behavioral symptoms. Moreover, PCEs were independently linked to fewer behavioral symptoms, underscoring their protective role in behavioral health (Bethell et al., 2019; Glickman et al., 2021). This beneficial influence of PCEs was further supported by Choi et al. (2024), who reported a significant association between PCEs and reduced internalizing and externalizing symptoms.

Integrating PCEs into resilience frameworks is particularly relevant to the challenge model. Moderate adversity, when buffered by PCEs, can serve as a developmental opportunity, teaching adaptive coping strategies and fostering psychological resilience (Kocatürk & Çiçek, 2023; Şanli et al., 2024). These findings underscore the importance of incorporating PCEs into prevention and intervention strategies, particularly in populations exposed to adversity. By focusing on enhancing PCEs, practitioners can help individuals not only recover from adversity but also achieve greater emotional, cognitive, and relational well-being (Bethell et al., 2019).

## Current Study

Previous systematic reviews on the relationship between PCEs and adult outcomes have found that higher levels of PCEs are associated with better mental health, behavior, and psychosocial functioning (Cunha et al., 2025; Han et al., 2023). However, these reviews also noted inconsistencies regarding the moderating and/or interaction effects of PCEs on the relationship between ACEs and adult outcomes. To date, there appears to be a lack of systematic reviews focusing specifically on the relationship between PCEs and various outcomes in children and adolescents. Therefore, this review aims to systematically examine and synthesize the literature on the relationship between PCEs and various outcomes in children and adolescents, identifying the most commonly studied outcomes. In addition, we aim to explore the effect of PCEs on these outcomes and to examine the potential interaction effects of PCEs on the relationship between ACEs and different outcomes in this population.

## Method

### Eligibility Criteria

The review followed the Preferred Reporting Items for Systematic Reviews and Meta-Analyses (PRISMA) guidelines (Page et al., 2021), and the protocol was pre-registered with OSF REGISTRIES (reference: 10.17605/OSF.IO/3EJPA) Studies were included if they met the following criteria: (a) were empirical research, (b) reported findings involving children and/or adolescents, (c) were written in English, Portuguese, or Spanish, (d) employed longitudinal or cross-sectional designs, and (e) examined the influence of PCEs on at least one outcome. Studies including juvenile or mixed samples (i.e., adults and juveniles) without separated statistical analysis were excluded. No restrictions were placed on publication dates.

### Search Strategy

In August 2024, the following electronic databases were searched: B-On, PsycINFO, PubMed, SCOPUS, and Scielo, using the search equation: (“positive childhood experiences” OR “benevolent childhood experiences” OR “childhood protective factors”) AND (impact OR consequence OR effect OR outcome OR influence). The search included no date restrictions and was conducted by two independent researchers. In addition, reference lists from included studies were also reviewed to identify additional relevant studies missed in the initial search.

### Literature Selection Process and Data Extraction

Studies identified through systematic searches were imported into Rayyan software (Ouzzani et al., 2016). Duplicates were removed, and titles and abstracts were screened based on the inclusion criteria for full-text analysis. A codebook was developed to extract data from the included papers, collecting information on references (e.g., authors and year), study characteristics (e.g., location and setting), sample characteristics (e.g., size, age, gender, and ethnicity/race), design characteristics (e.g., design type and follow-up duration), measurements (e.g., assessment tools), and outcomes. Two independent raters conducted the screening and data extraction phases. Discrepancies between raters were resolved through discussion, with a third reviewer available.

### Quality Assessment

The methodological quality of all included studies was assessed using the Mixed Methods Appraisal Tool (MMAT; Hong et al., 2018). This tool begins with two screening questions: “Are there clear research questions?” and “Do the collected data allow to address the research questions?” It then evaluates the methodological quality through five items specific to the study design. Each item is rated as “yes,” “no,” or “can’t tell.”

## Results

### Screening and Selection of Studies

**Figure 1.**
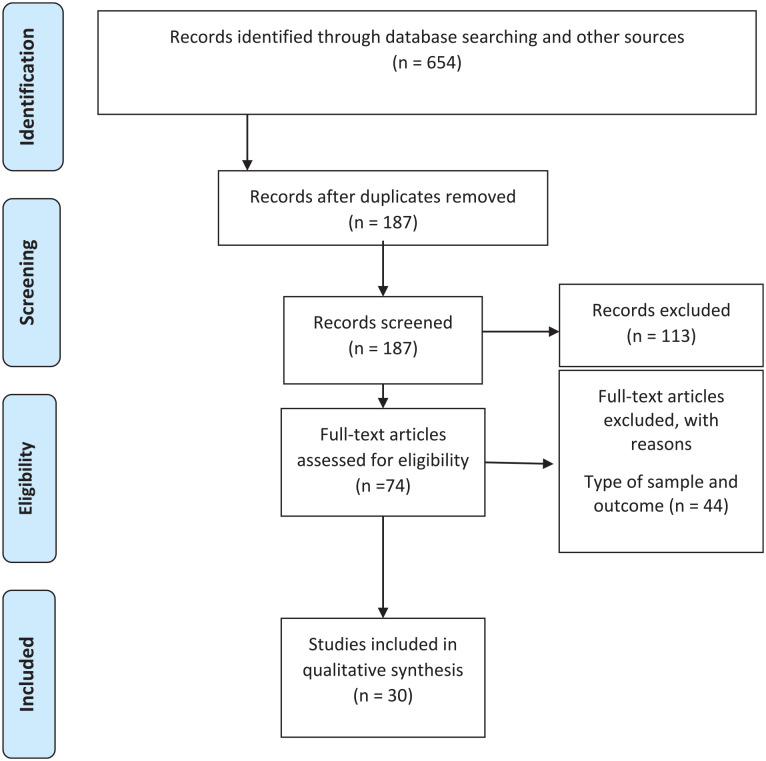
PRISMA flow diagram of the study selection process.

In total, 654 articles were identified through data searches and supplementary searches. After removing duplicates, 187 titles, and abstracts remained and were screened for relevance. A total of 74 studies were selected for further examination. However, 44 additional studies did not meet the inclusion criteria, primarily because they involved both juvenile and adult individuals and did not report relevant outcomes. Ultimately, 30 studies were included in the final review. Figure 1 presents the PRISMA flow diagram, illustrating the number of studies included at each stage of the selection process and the reasons for exclusion.

### Quality Assessment

Among the included articles, most were designed as quantitative non-randomized studies (*n* = 25), and the remaining conducted quantitative descriptive studies (*n* = 4) and quantitative randomized studies (*n* = 1; see Table 1).

**Table 1.** Main Effects of Positive Childhood Experiences (PCEs).

Study	Sample (ethnicity)	PCEs Measure	Outcomes	Findings
Anderson et al. (2024)	35,583 (n/a)	Self-constructed	MH	Some PCEs were associated with a lower prevalence of anxiety, depression, and behavioral or conduct problems. Results typically remained consistent even after accounting for ACEs.
Bunting et al. (2023)	1,299 (n/a)	BCEs	MH	Each additional PCE decreased the likelihood of common mood and anxiety disorders, self-harm, and suicidal ideation. PCEs did not moderate the relationship between ACEs and mental health outcomes.
Choi et al. (2024)	10,915 (53.9% non-Hispanic White; 15.6% Hispanic, 14.9% non-Hispanic Black)	Self-constructed	MH	PCEs were associated with clinical-range externalizing and internalizing symptoms (anxiety, depression, somatization, aggression, attention problems, and oppositional behaviors). Still, these associations were non-significant once other ACEs were added to models. Only one PCE was associated with clinical-range scores, even when accounting for other ACEs.
Craig et al. (2022)	23,985 (38.3% White; 46.2% Black; 15.5% Hispanic)	Self-constructed	BO	Individuals with low ACEs and high PCEs had 27.5% lower rearrest rates.
Craig et al. (2021)	91,316 (White: 35.50%; Black 48.60%; Latinx 15.40%; Other 0.50%)	Self-constructed	MH; SDF; BO	Being black was strongly associated with fewer PCEs while being male was positively associated with PCEs. Behavioral problem diagnosis, impulsivity, and substance abuse were negatively correlated to cumulative PCE exposure.
Crouch, Radcliff, et al. (2022)	28,771 (51.1% Non-Hispanic white, 13.3% Non-hispanic black; 25.6% Hispanic; 9.9% Other)	Self-constructed	PH	PCEs were not significantly associated with overweight or obesity in children who had experienced zero to one ACE.
Crouch, Smith & Andersen et al. (2022)	33,450 (51.8% Non-Hispanic white; 13.1% Non-Hispanic African American; 24.9% Hispanic; 10.2% other)	HOPE	Other	Children exposed to household incarceration were less likely to experience some PCEs.
Crouch et al. (2021)	33,450 (non-Hispanic white: 51.8%; Non-Hispanic African American: 13.1%; Hispanic: 24.9%: “Other” Non-Hispanic: 10.2%)	Self-constructed	AAO	Children with school absenteeism were less likely to experience each type of PCE than their counterparts, except for volunteerism and mentorship. Children who repeated a grade were less likely to report exposure to each type of PCE than their peers, except for living in a safe and supportive neighborhood.
Couch et al. (2023)	63,000 (non-Hispanic white: 52.9%; Non-Hispanic African American: 13.1; Hispanic: 24.5; NH American Indian/Alaska Native: 4.4; NH Asian/Pacific Islander: 0.4; Other: 4.7)	Self-constructed	SDF	Rural children were more likely than urban children to be reported as having PCEs.
Deng et al. (2023)	1,706 (n/a)	BCEs	PO	Resilience partially mediated the impact of peer victimization on adolescents’ quality of life, with PCEs moderating this mediation.
Dubow et al. (2016)	436 (90% Caucasian)	Self-constructed	BO	Exploring protective factors at age 8, some PCEs of the parents reduced the risk of violence in adulthood. For at-risk males, an increase in the number of protective factors at age 8 did not significantly change the chances of becoming a violent adult. For males with no risk factors, an increase in the number of age 8 protective factors substantially decreased the likelihood of becoming a violent adult.
Duncan et al. (2019)	1,169 (50% African American; 28% Hispanic; 20% Other)	Self-constructed	BO; MH	Positive Action was related to changes in substance use and violence from late childhood to early adolescence.
Elmore et al. (2020)	40,302 (52.3% Non-hispanic white; 13.3% non-hispanic black; 24.5% Hispanic; 9.9% Other)	Self-constructed	MH	There were associations between depression and low resiliency, as well as significant interactions between ACE exposure and three PCEs.
Guo et al. (2021)	3,111 (85.8% Anglo-European; 11.8% ethnic minority; 2.5% indigenous)	Self-constructed	MH; AAO	Higher exposure to PCEs across each domain was associated with lower reporting of mental health problems and academic difficulties in adolescence.
Hinojosa et al. (2023)	22,628 (Hispanic/Latino 25.21%; Non-Hispanic/Latino 74.79%)	Self-constructed	MH	For each one-unit increase in PCEs, the odds of reporting a mental health condition are 24 % lower. PCEs moderate the relationship between ACEs and reports of mental health conditions.
Johnson et al. (2022)	1,098 (73.86% Caucasian)	BCEs	MH	Regardless of the extent of PCEs, children who reported moderate-to-high levels of ACE faced an increased risk of mental health difficulties.
Kosterman et al. (2011)	429 (98% white)	Self-constructed	PO; MH	PCEs predicted significantly better functioning for each model, even after accounting for adolescent substance use, and consistently predicted significantly less adolescent substance use.
Kowalski et al. (2022)	254,874 (55.9% white; 35% black; 5.6% hispanic or latino; 3.5% other)	Self-constructed	BO	The presence of PCEs was linked to a reduced likelihood of reoffending, whereas ACEs were associated with higher odds of recidivism. PCEs demonstrated a protective impact on ACEs.
Luo et al. (2024)	6,982 (n/a)	BCEs	PO	PCEs were associated with higher health-related quality of life.
Mueller et al. (2023)	3,604 (44.48 % Black, 46.39 % Hispanic, 8.38 % White, and 0.75 % Other)	Self-constructed	PO	A greater number of PCEs is associated with future orientation.
Novak and Fagan, (2022)	1,354 (27% white, 53% black, 13% other race, 7% hispanic)	Self-constructed	BO	PCEs were associated with significantly fewer delinquent acts but no arrests. PCEs significantly reduced the relationship between ACEs and delinquency but only for youth with moderate levels of ACEs.
Priest et al. (2022)	3,488 (89.0% Anglo-European/White, 9.7% ethnic minority/non-white, 1.3% Indigenous, 4.1% non-White)	Self-constructed	PH	Small but directionally consistent associations exist between adverse experiences and higher inflammation and positive experiences and lower inflammation.
Pugh et al. (2023)	47,514 (51.2% white)	Self-constructed	PH	Children reporting the lowest number of PCEs had a prevalence of chronic pain that was twice as high as those reporting the highest number of PCEs.
Qu et al. (2022)	6,363 (n/a)	Self-constructed	MH	PCEs were negatively associated with the risk of depression and anxiety.
Rhodes et al. (2023)	19,120 (non-Latinx White: 70%; 6% Black/ African American; 5% Asian; 1% Native American or Alaskan, <1% Native Hawaiian or Pacific; 3% “Other”, 7% multiracial; 12% Latinx/Hispanic)	HOPE	MH	PCEs were associated with lower odds of mental health problems for both children with and without incarcerated parents. However, PCEs did not mitigate the negative impact of parental incarceration on mental health outcomes.
Samji et al. (2024)	8,864 (3% Black; 18% East Asian; 11% Indigenous; 4% Latin American; 5% Middle Eastern; 8%, South Asian; 8% Southeast Asian; 60%, White, 4% Another Race)	Self-constructed	MH; PO	Six or more PCEs were associated with better mental health and well-being in adolescents with and without ACEs. PCEs significantly moderated the association between ACEs and depression.
Wang et al. (2021)	3,426 (20.7% Non-Hispanic White; 50.1% Non-Hispanic Black; 25.9% Hispanic, 3.3% other)	Self-constructed	MH	PCEs were associated with lower odds of depression and anxiety.
L. Zhang et al. (2021)	1,809 (n/a)	Self-constructed	MH	PCEs can lessen the adverse impact of prolonged childhood maltreatment on adolescent mental health, with greater PCEs leading to better outcomes.
X. Zhang et al. (2023)	11,4709 (White (68.92%, Hispanic 12.17%, Other; 7.88%; Asian 4.·96%)	Self-constructed	Other	There is an inverse relationship between food insecurity and the rate of PCE across all age categories.
Zhu et al. (2023)	2,587 (n/a)	BCEs	PO	Children whose mothers reported high PCE scores were less likely to have psychosocial challenges. PCEs slightly neutralized the negative effects of ACEs on offspring’s total difficulties and prosocial problems.

*Note.* MH = Mental Health; SDF = Sociodemographic factors; BO = Behavioral Outcome; PH = Physical Health; PO = Psychosocial outcomes; AAO = Academic Achievement Outcome; HOPE = Health Outcome Positive Experiences (HOPE).

Of the included studies, two met all five criteria of excellence (Choi et al., 2024; Elmore et al., 2020), four met four out of five criteria (Crouch, Radcliff, et al., 2022; Crouch et al., 2023; Luo et al., 2024; Pugh et al., 2023), nine fulfilled three out of five criteria (Anderson et al., 2024; Craig et al., 2021; Crouch, Smith & Andersen, 2022; Hou et al., 2022; Kosterman et al., 2011; Qu et al., 2022; Rhodes et al., 2023; X. Zhang et al., 2023; Zhu et al., 2023), 13 presented two out five criteria (Bunting et al., 2023; Crouch et al., 2021; Craig et al., 2022; Deng et al., 2023; Duncan et al., 2019; Dubow et al., 2016; Hinojosa et al., 2023; Johnson et al., 2022; Kowalski et al., 2022; Priest et al., 2022; Novak & Fagan, 2022; Samji et al., 2024; L. Zhang et al., 2021), and two met only one out five criteria (Guo et al., 2021; Wang et al., 2021). The major limitations of the studies are related to the failure to include a representative sample of the population, the lack of consideration for confounding factors in the analysis, the use of inappropriate instruments to assess the outcome, and the inability to obtain complete outcome data.

### Characteristics of Included Studies

#### Study Characteristics

The publication years of the included papers ranged from 2011 (Kosterman et al., 2011) to 2024 (Anderson et al., 2024; Choi et al., 2024; Luo et al., 2024; Samji et al., 2024): 2011 (*n* = 1), 2016 (*n* = 1), 2019 (*n* = 1), 2020 (*n* = 1), 2021 (*n* = 5), 2022 (*n* = 9), 2023 (*n* = 8), and 2024 (*n* = 4). More than half of the studies (*n* = 20) were conducted in the USA, followed by five in China, two in Australia and Canada, and one in the UK. Table 1 summarizes the key characteristics of the studies included.

#### Sample Characteristics

The studies included in the analysis varied widely in sample sizes, with participant numbers ranging from 429 individuals (Kosterman et al., 2011) to 254,874 individuals (Kowalski et al., 2022). Participants’ ages spanned from 0 years (Wang et al., 2021) to 18 years (Johnson et al., 2022). Additionally, some studies reported the average age of the participants, which ranged from 5.35 years (Zhu et al., 2023) to 16.29 years (Novak & Fagan, 2022).

Almost all the studies were conducted in community settings, with the exception of four that recruited participants from forensic settings (Craig et al., 2021, 2022; Kowalski et al., 2022; Mueller et al., 2023). Similarly, nearly all samples included both men and women, except for one study that focused exclusively on male individuals (Dubow et al., 2016) (see Table 1). Among the studies presented, ethnicity/race varies substantially. The percentage of non-Hispanic White individuals ranges from 20.7% (Wang et al., 2021) to 53.9% (Choi et al., 2024), Hispanic individuals from 7% (Novak & Fagan, 2022) to 46.39% (Mueller et al., 2023), and non-Hispanic Black individuals from 13.3% (Crouch, Smith & Andersen, 2022; Elmore et al., 2020) to 50.1% (Wang et al., 2021). Moreover, the percentage of White individuals ranges from 8.38% (Mueller et al., 2023) to 98% (Kosterman et al., 2011) and Black individuals from 3% (Samji et al., 2024) to 53% (Novak & Fagan, 2022). The remained studies included other ethnicities, namely three studies with a minor percentage of Non-Hispanic African American (13.1%) (Crouch et al., 2021; Couch, Radcliff, et al., 2022; Couch et al., 2023), another study with Non-Hispanic Indian/Alaska Native (4.4%; Couch et al., 2023), another with a residual percentage of Non-Hispanic Pacific Islander (0.4%; Couch et al., 2023), and another with a higher percentage of Caucasian (Dubow et al., 2016; Johnson et al., 2022). Moreover, seven studies did not report information about ethnicity.

#### PCEs measures

Most studies (*n* = 17) used adaptations of preexisting scales or screening questions to measure PCEs, while the remaining studies (*n* = 5) used the BCEs scale (Narayan et al., 2018).

#### Outcomes

Mental health issues were the most frequently examined outcomes (*n* = 15), followed by psychosocial (*n* = 6) and behavioral outcomes (*n* = 6). In addition, a few studies focused on physical health (*n* = 3), academic achievement (*n* = 2), sociodemographic factors (*n* = 2), and other outcomes (*n* = 2).

The outcome categories in this systematic review were defined based on previous reviews (Cunha et al., 2025; Han et al., 2023). “Mental health problems” refer to clinical psychological issues, such as mood disorders and/or substance use problems. “Psychosocial outcomes” encompass non-clinical psychological factors (e.g., self-esteem, well-being, gratitude, and perceived stress) and external resources (e.g., social support) that influence individuals' well-being and social functioning. “Behavioral outcomes” relate to criminal delinquency and aggressive behavior, while “physical health outcomes” include various physical health indicators. Lastly, “academic achievement outcomes” refer to all types of academic performance outcomes (e.g., academic difficulties).

The studies assessed a variety of mental health outcomes, including mood disorders (e.g., anxiety and depression) (*n* = 7), substance use (*n* = 3), behavioral disorders (*n* = 3), somatization (*n* = 1), attention problems (*n* = 1), suicidal ideation (*n* = 1), and self-harm (*n* = 1). Furthermore, six studies examined mental health problems without specifying particular psychiatric symptoms or disorders. Among studies focusing on psychosocial problems, the assessed areas included quality of life (*n* = 3), adult functioning (*n* = 1), future orientation (*n* = 1), and psychosocial issues (*n* = 1). For behavioral outcomes, the primary areas of interest included rearrest/reoffending rates (*n* = 3), violence (*n* = 2), delinquent acts (*n* = 1), and impulsivity (*n* = 1). In other categories, physical health outcomes included obesity/overweight (*n* = 1), inflammation (*n* = 1), and chronic pain (*n* = 1), while academic achievement outcomes focused on absenteeism (*n* = 1) and academic difficulties (*n* = 1).

##### Mental Health

All studies reported that higher PCEs predicted lower rates of depressive symptoms, anxiety, self-harm, behavioral disorders, somatization, attention problems, substance use, and suicidal ideation (see Table 1). However, inconsistencies emerge regarding the ability of PCEs to mitigate the effects of ACEs. Bunting et al. (2023), Johnson et al. (2022), and Samji et al. (2024) found that PCEs did not moderate the relationship between ACEs and mental health outcomes. In contrast, Hinojosa et al. (2023) and L. Zhang et al. (2021) reported evidence supporting this moderating effect. In addition, one study found that only one specific PCE—cumulative discrimination—was associated with clinical problems, even when accounting for ACEs (Choi et al., 2024), and another study found that the mental health outcomes remained consistent even after accounting for ACEs (Anderson et al., 2024).

##### Psychosocial Outcomes

The studies revealed that higher PCEs were associated with better functioning, future orientation (i.e., the internalization of hope and optimism regarding potential outcomes), and improved quality of life/well-being (Luo et al., 2024; Samji et al., 2024). In adddition, one study focusing on mothers and their children, and the intergenerational component, reported that children whose mothers had high PCE scores were less likely to experience psychosocial challenges (Zhu et al., 2023). Studies focused on the relationship between ACEs and PCEs consistently reported that PCEs moderate the relationship between ACEs and quality of life (Deng et al., 2023) and prosocial problems (Zhu et al., 2023).

##### Behavioral Outcomes

Almost all studies reported that higher PCEs predicted lower rearrest/reoffending rates (Craig et al., 2022; Kowalski et al., 2022), reduced violence (Dubow et al., 2016; Ducan et al., 2019), lower impulsivity (Craig et al., 2021), and fewer offending acts (Novak et al., 2022; see Table 1). However, Novak et al. (2022) found that PCEs were not associated with fewer arrests. Furthermore, Dubow et al. (2016) examined the intergenerational component and concluded that certain PCEs, such as parents attending church and experiencing fewer negative family interactions, reduced the risk of violence. In addition, PCEs were shown to have a protective effect on ACEs (Kowalski et al., 2022), but this was only true for youth with moderate levels of ACEs (Novak et al., 2022).

##### Other Outcomes

Higher PCEs were associated with lower levels of inflammation and chronic pain, but no association was found with obesity/overweight (Crouch, Radcliff, et al., 2022; Priest et al., 2022; Pugh et al., 2023). Additionally, higher levels of PCEs were linked to less school absenteeism and fewer academic difficulties (Crouch et al., 2021; Guo et al., 2021). Regarding sociodemographic factors, boys exhibited more PCEs than girls, while Black children had fewer PCEs than non-Black children (Craig et al., 2021). However, this difference disappeared when accounting for the neighborhood context. Moreover, rural children had more PCEs than their non-rural counterparts (Crouch et al., 2023). Two studies, which do not fit into any specific category, presented the following findings: one study found that children exposed to household incarceration were less likely to experience certain PCEs (Crouch, Smith, & Andersen, 2022), while another study revealed an inverse relationship between food insecurity and the rate of PCEs (X. Zhang et al., 2023).

## Discussion

Childhood and adolescence are critical developmental stages that profoundly influence individuals’ overall growth and development. According to developmental psychology, these formative years are essential for shaping cognitive, emotional, and social competencies (Branje et al., 2022; Pozzi et al., 2021). Consequently, it is imperative to understand the influence of PCEs during these stages. Understanding the mechanisms by which PCEs buffer or interact with ACEs is particularly important, as these insights can inform interventions aimed at mitigating the effects of childhood adversity. Understanding the developmental origins of inequalities in well-being, as indicated by positive functioning, is key to ensuring equitable life chances throughout the life course. Therefore, this systematic review aims to synthesize the existing literature on PCEs and evaluate their effects on positive and negative outcomes, with a focus on children and adolescents. Although some systematic reviews are focusing on the effects of PCEs in adults (Cunha et al., 2025; Han et al., 2023), this is, to the best of our knowledge, the first systematic review to provide a comprehensive perspective on the effects of PCEs across multiple outcome categories (e.g., mental health and psychosocial outcomes) with samples of children and adolescents.

Our results revealed that nearly all the studies included were published within the last 5 years, reflecting the growing interest of the scientific community in PCEs, which is consistent with the findings from previous reviews (e.g., Cunha et al., 2025; Han et al., 2023). The interest stems from the need to prevent the adverse effects of childhood experiences—factors first studied in the pioneering work of Felitti et al. (1998)—and to understand the protective factors that may mitigate the harmful outcomes of ACEs (Shonkoff et al., 2012). Research has shown that both ACEs and PCEs can influence brain development and health throughout life (Shonkoff et al., 2012). Despite this growing interest, the focus on children and adolescents has not been as prominent. The number of publications included in this review is significantly lower compared to two systematic reviews on the same topic but mostly with adults (Cunha et al., 2025; Han et al., 2023).

At the same time, research on PCEs remains diverse and inconsistent across different countries and settings. Nearly all studies were conducted in the United States and within community settings, with only one study conducted in a European country (i.e., the UK). Furthermore, only a small number of studies included participants from forensic settings, and none included clinical samples. Variability in cultural and sociodemographic contexts can influence how PCEs and ACEs interact, as resilience models consider adaptation to social and cultural contexts. Future studies should explore how different cultures and populations experience and respond to adversity, which would help refine resilience models.

These findings highlight a disparate interest in the studying of PCEs, which is surprising given that ACEs and PCEs can vary significantly across culturally diverse populations (e.g., Merrick et al., 2020) and social contexts (e.g., Wang, 2023). In addition, previous studies involving adult individuals have shown that individuals in forensic (e.g., Astridge et al., 2023; Hilton et al., 2019) and clinical settings (e.g., Madigan et al., 2023) tend to report higher levels of ACEs compared to community samples. This underscores the importance of identifying PCEs in these populations to better understand the interactional effects of PCEs (Cunha et al., 2025). Our findings also reveal that most studies lack a standardized protocol for assessing PCEs, often relying on measures developed specifically for individual investigations or screening questions. This limitation may be partly attributed to the relatively recent development of the Benevolent Childhood Experiences Scale (Narayan et al., 2018), the most commonly used instrument in the field.

Most studies included in this systematic review examined the impact of PCEs on mental health outcomes, demonstrating that PCEs were associated with few mental health problems (e.g., depressive symptoms, anxiety, self-harm, behavioral disorders, somatization, attention problems, substance use, and suicidal ideation). These results align with previous systematic reviews focusing on adult samples (e.g., Cunha et al., 2025; Han et al., 2023) and suggest that PCEs have an independent effect on mental health outcomes. While results related to other outcomes, such as psychosocial and behavioral problems, generally indicate a positive effect of PCEs, further investigation is needed due to the limited number of studies examining each specific outcome. Nonetheless, these findings align with theoretical perspectives suggesting that PCEs exert a significant influence on brain development (Luby et al., 2019; Masten & Barnes, 2018; Op den Kelder et al., 2018). This influence is particularly relevant for the development of self-regulation and executive functioning skills, which are critical for long-term health and well-being (e.g., Zelazo & Carlson, 2020). These perspectives emphasize the potential buffering role of PCEs, particularly in fostering self-regulation and executive functioning, which are critical for mitigating the long-term effects of ACEs.

Furthermore, areas such as physical health, academic performance, sociodemographic factors, and other related outcomes have received comparatively less attention. For instance, only a small number of studies have examined the impact of PCEs on school outcomes (e.g., Crouch et al., 2021; Guo et al., 2021). Given that ACEs are strongly associated with poor educational achievements (e.g., Stewart-Tufescuet al., 2022), and that early childhood adversities can profoundly impair academic trajectories for children and adolescents (e.g., Boullier & Blair, 2018; Dube et al., 2006), this lack of research restricts our understanding of potential benefits of PCEs in educational domains. This gap underscores the need for further studies to explore how PCEs influence educational outcomes and other critical areas. In addition, research indicates that male and non-Black juveniles report higher levels of PCEs compared to female and Black children (Craig et al., 2021). These findings align with existing literature, which indicates that females (e.g., Baglivio et al., 2014; Jones et al., 2022) and Black juveniles (e.g., Sacks & Murphey, 2018; X. Zhang & Monnat, 2021) face greater risks of experiencing ACEs than their male and non-Black counterparts. The inverse relationship between ACEs and PCEs has been well-documented (e.g., Almeida et al., 2021; Crouch, Radcliff, et al., 2022), suggesting that higher exposure to ACEs is often associated with lower levels of PCEs. However, this association underscores the importance of examining how factors such as systemic inequalities and neighborhood social disorganization mediate these relationships, as these variables may disproportionately affect marginalized populations (e.g., Craig et al., 2021). Despite these insights, the limited body of research examining these outcomes hinders the ability to draw more definitive conclusions. This highlights the critical need for further investigations to deepen our understanding of the nuances and effects of PCEs across diverse populations and contexts.

Despite the documented impact of PCEs on various outcomes, evidence regarding their role as a buffer against the effects of ACEs remains inconclusive. Specifically, research has shown inconsistent moderating and interaction effects of PCEs on the presence of ACEs (e.g., Anderson et al., 2024; Bunting et al., 2023; Deng et al., 2023; Johnson et al., 2022; Samji et al., 2024; Zhu et al., 2023). According to resilience models, which emphasize the dynamic interaction between risk factors (ACEs) and protective factors (PCEs), positive experiences can serve as buffers, reducing the cumulative impact of childhood adversity and promoting positive developmental outcomes (Andersson & Ledogar, 2008; Bunting et al., 2023). However, these inconsistencies may stem from variations in measurement tools, the inherent nature of PCEs, and the complexity of the interactions with ACEs. One major challenge lies in the high variability of instruments used to assess PCEs. This results in a wide range of childhood experiences being categorized as positive across studies, potentially leading to differing outcomes based on the specific experiences considered. In addition, PCEs have a cumulative effect, with their benefits increasing as multiple positive experiences build up across various domains (Han et al., 2023). This accumulation further enhances their protective capacity. This process may explain their effectiveness in buffering the adverse effects of ACEs. This concept aligns with resilience models, which emphasize that the accumulation of positive experiences over time can foster recovery and adaptation in the face of adversity (Andersson & Ledogar, 2008; Bunting et al., 2023). Therefore, the more experiences and levels of PCEs considered, the greater their potential impact on outcomes, especially in the presence of ACEs. In this context, examining PCEs across different levels of ecological systems (e.g., microsystem, mesosystem, exosystem, macrosystem, and chronosystem; see Bronfenbrenner, 1977) may provide more reliable insights. The cumulative nature of PCEs underscores their significance in fostering favorable developmental trajectories (Crandall et al., 2019; Evans et al., 2013; Masten & Cicchetti, 2016; Masten et al., 2021). By utilizing an ecological framework, future research can better capture the multifaceted ways in which PCEs operate across systems to influence child and adolescent development. Furthermore, the method of assessing PCEs—whether as a continuous variable or merely as the presence or absence of such experiences—may also influence observed effects, particularly regarding their potential buffering role in the context of ACEs. This distinction is crucial, as research suggests a dose-response relationship between PCEs and adult mental health, similar to the cumulative effects of multiple ACEs (Bethell et al., 2019). Moreover, cultural differences may also contribute to these inconclusive results, as the included studies are diverse in this regard. Research further demonstrates that not all ethnic groups exhibit the same prevalence of ACEs (Madigan et al., 2023). Lastly, the severity of ACEs may also explain these inconclusive results, given the cumulative effect these experiences can have (Madigan et al., 2023). Therefore, future studies should address the effects of measurement variability, the intrinsic nature of PCEs and ACEs, the cultural differences associated with these experiences and their cumulative effects to better understand the role shaping experiences.

### Limitations

Although the methods employed in this systematic review provided a thorough analysis of the existing literature, there are limitations that need to be acknowledged. This review was restricted to studies published in English, Portuguese, and Spanish to ensure accurate interpretation by fluent authors. However, this exclusion of potentially relevant papers in other languages may have influenced the findings of the review. Furthermore, while no restrictions were applied regarding the research setting or country, most of the included studies are quite homogeneous in these variables (with the majority conducted in community settings and the United States). This homogeneity limits the generalizability of the findings to other countries and locations. Therefore, future research should aim to include more diverse samples, considering various cultural and contextual backgrounds as well as different settings (such as clinical and forensic environments) to gain a clearer understanding of the specific characteristics of PCEs and their potential protective or promotive roles. In addition, the small number of studies addressing behavioral, psychosocial, physical health, and academic achievement outcomes, along with inconsistent findings in some areas, hinders the ability to reach definitive conclusions. Future research should explore the impact of PCEs on these outcomes more thoroughly. Lastly, some limitations in the included studies regarding bias should also be considered, and a careful analysis should be conducted. Therefore, future studies should employ an appropriate methodology and include representative samples.

### Conclusions and Implications

There has been a recent surge of interest in the impact of PCEs on children and adolescents. However, research on younger populations remains comparatively limited, with a smaller number of publications than the extensive body of studies focusing on adults (e.g., Cunha et al., 2025; Han et al., 2023). Despite some inconsistencies in the findings, these studies highlight the importance and need of considering PCEs in the broader context of child and adolescent development, emphasizing the promotion of these experiences as a key strategy in preventing the harmful effects of ACEs (see Table 2).

**Table 2.** Key Findings of the Systematic Review.

Key Findings
Most studies were published within the last four years, in the USA.
Research primarily focused on community populations.
Higher levels of PCEs were linked to improved mental health, enhanced psychosocial outcomes, better academic performance, and some positive effects on physical health.
The relationship between PCEs and behavioral outcomes showed mixed results.

Firstly, it is crucial to advance efforts aimed at reducing negative outcomes in childhood/adolescence, emphasizing the importance of greater public health focus on evaluating and promoting PCEs and broader child/adolescence resilience resources. A combined assessment of ACEs and PCEs can enhance efforts to evaluate needs, target interventions, and address adversity by harnessing the strengths of children and families. Given the protective nature of PCEs, interventions for children and adolescents at risk of ACEs should prioritize fostering positive experiences in family, school, and community settings. By enhancing the presence of PCEs in these environments, we can more effectively mitigate the long-term effects of adversity.

Secondly, families/communities/clinical and educational services, specifically those working with children at risk and who live without their families, should focus on promoting the integration of multiple PCEs, which together foster resilience and improve lifelong health. The literature indicates that resilient functioning arises from a combination of promotive and protective factors across different levels of individual functioning—biological, psychological, and social. Therefore, possessing a range of skills and resources across these levels of interaction is more critical for long-term health and functioning than focusing on a single positive experience (Crandall et al., 2020). In fact, some institutions have suggested policies and programs that include strengthening positive interactions between children living in disadvantaged environments and the adults who care for them (e.g., National Scientific Council on the Developing Child, 2015).

Future research should focus on more diverse samples, include varied cultural contexts, and more deeply explore the impacts of PCEs on outcomes beyond mental health, including psychosocial, behavioral, and academic outcomes. This will enable a more holistic understanding of the benefits of PCEs and contribute to the development of more effective interventions to support the well-being of children and adolescents in different contexts (see Table 3).

**Table 3.** Implications for Research, Practice, and Policy.

Implications
The findings advocate for expanding resources to build resilience in children and adolescents.
Families, communities, and support services should focus on integrating multiple PCEs.
More diverse samples that include varied cultural contexts and more in-depth exploration of the impacts of PCEs on outcomes beyond mental health, such as psychosocial, behavioral, and academic outcomes, are needed.
